# Echo-Doppler And Electrogram Correlation: A Guide For The Invasive Management Of Patients With Atrial Fibrillation

**Published:** 2009-07-01

**Authors:** Kaniz Fatema, James B Seward, Samuel J Asirvatham

**Affiliations:** 1 Division of Cardiovascular Diseases, Mayo Clinic, Rochester, Minnesota; 2 Department of Pediatrics and Adolescent Medicine, Mayo Clinic, Rochester, Minnesota

**Keywords:** Doppler, atrial fibrillation, ablation

Atrial fibrillation is a multivariable disease  [1]. It is generally considered that in young patients without cardiac abnormalities AF may represent a primary electropathy with triggers in the pulmonary vein [2]. Whereas in older patients with structural heart disease left atrial enlargement ascribed to chronic pressure overloads is thought to cause a secondary substrate abnormality allowing the propitiation of this arrhythmia. Despite this simplistic paradigm much overlap exists with some patients in the former group failing to benefit from ablation therapy (expected to be successful without substrate abnormalities) and others with severe atrial enlargement unexpectedly responding well to ablation. In this pictorial report we describe the association of echo Doppler based diastolic function data being an accurate predictor of primary electropathy and thus benefit from ablation procedures [3].

## Case 1

**Figure 1:**
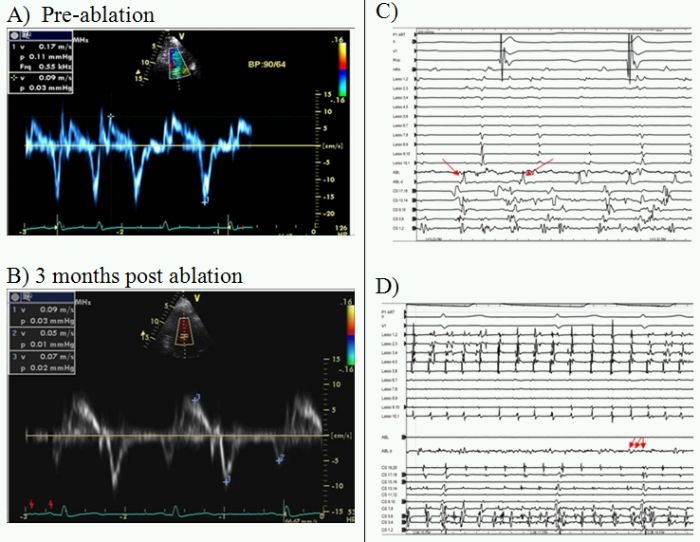
**A** pre- and **1B** post-ablation tissue Doppler velocity tracings obtained on septal mitral annulus showing normal diastolic function (tissue Doppler e' > 10 cm/s).  The electrograms **IC** pre and post **ID** ablation show initial excellent amplitude electrograms (arrows) fragmented only with ablation suggesting fundamentally healthy atrial tissue as suggested by the normal tissue Doppler findings (primary electropathy).

39 year healthy male with 20 year history of atrial fibrillation (AF).  Left ventricular systolic function was reduced (LVEF 30%). Despite long-standing AF and structural heart disease pulmonary vein isolation resulted in excellent benefit with subsequent normalization of EF (56% at 3 months follow up) (Figure 1).

## Case 2

**Figure 2:**
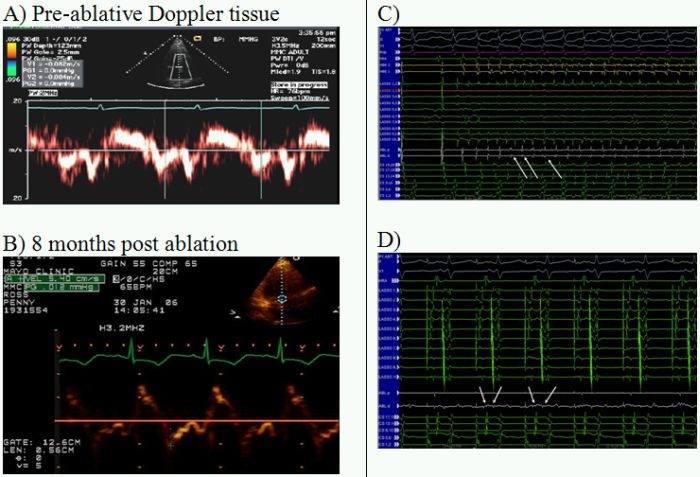
**A** pre-ablation Doppler velocity tracings at the septal mitral annuls show mild diastolic dysfunction (tissue Doppler e' > 8 cm/s).  Eight months post-ablation (**B**) showed continued reduced diastolic function (e' 5 cm/s) but corrected volume overload with LAVI decreased from 22 ml/m^2^ from pre-ablation value 33 ml/m2. **C/D** show pre-ablation electrograms from the left atrium close to the pulmonary vein ostium.  Amplitude is lower and some fragmentation is seen showing early evidence of substrate involvement.

52-year-old female with diabetes, obesity and paroxysmal AF.  Systolic ventricular function was normal. Radiofrequency ablation was required to isolate the pulmonary veins, as well as additional focal sources of arrhythmia in the superior vena cava and coronary sinus (Figure 2).

## Case 3

**Figure 3:**
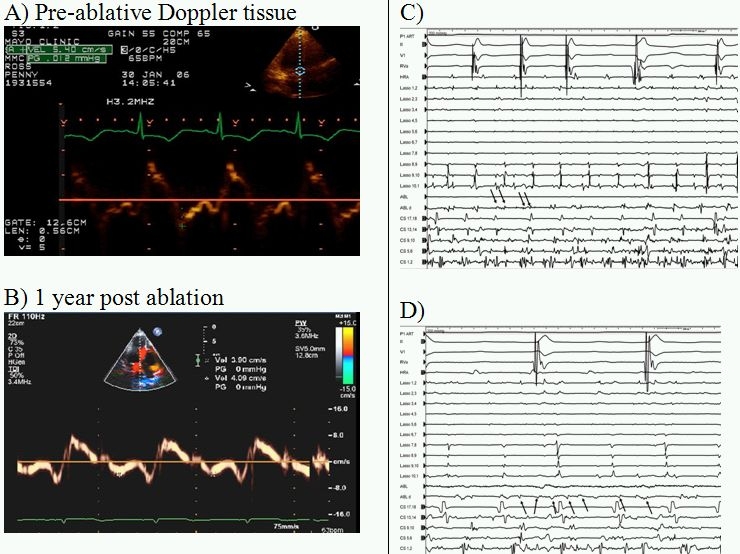
**A** pre-ablation Doppler velocity tracing showing moderate diastolic dysfunction (e' cm/s).  Echo parameters were essentially unchanged 1 year post ablation **B** (e' 4 cm/s and LAVI 72 ml/m^2^).  **C/D** show pre- and post-ablation electrograms with varying degrees of organization during persistent fibrillation and highly fragmented signals that correlated with echo parameters signifying advanced secondary atrial disease.

60-year-old male with persistent atrial fibrillation, history of nonobstructive hypertrophic cardiomyopathy, moderate diastolic dysfunction and severe left atrial enlargement (LAVI 75 ml/m^2^). Despite pulmonary vein isolation multiple atrial flutters were noted necessitating extensive liner ablation with eventual control that required pace termination and adjunctive medical therapy (Figure 3).

## Case 4

**Figure 4:**
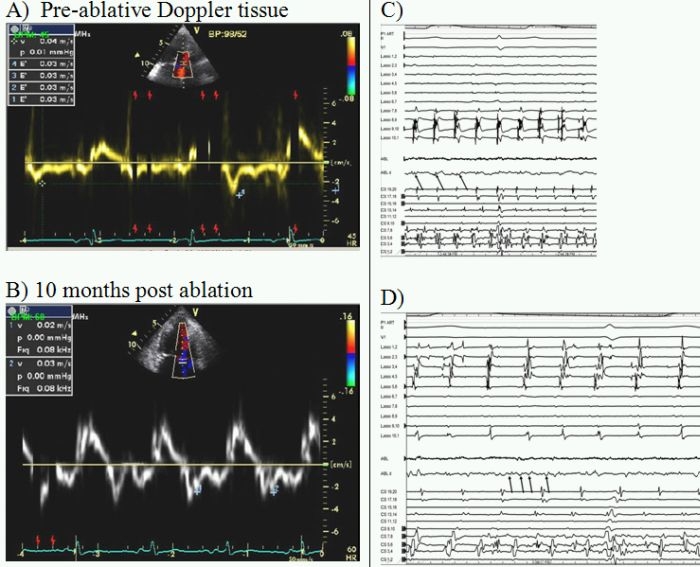
**A** pre-ablation tissue Doppler velocity increasing showing severe diastolic dysfunction (e' 3 cm/s) findings were virtually unchanged 10 months post ablation (**B**) with e' 3 cm/s,  LAVI of 82 ml/m^2^ and LVEF 44%. **C/D** show representative intracardiac atrial electrograms pre- and post-ablation respectively.  Marked electrogram fragmentation with high frequency signals suggesting of advanced secondary atrial substrate disease that correlated with echo based prediction.

56-year-old with chronic atrial fibrillation restrictive cardiomyopathy and moderate tricuspid regurgitation. Echo exam showed severe diastolic dysfunction and marked atrial enlargement (LAVI 73 ml/m^2^ and LVEF 45%). Despite wide-area circumferential ablation around the pulmonary veins and adjunctive medical therapy a modified right and left sided endocardial maze procedure control of arrhythmia has been suboptimal with subsequent requirement of permanent pacemaker implantation (Figure 4).

## Summary

A collage of four cases demonstrating a spectrum of correlation between echo Doppler variables and intracardiac electrograms that accurately defined the electropathy and predicted outcomes of ablation are presented.  Further studies will help define whether echo based assessment of diastolic function will help guide patient choice for invasive electrophysiology procedures.

## Abbreviations

AF: Atrial fibrillation
e': myocardial velocity
EF: ejection fraction
DCCV: direct current cardioversion
LBBB: left bundle branch block
LAVI: left atrial volume index
LV: left ventricle
LVEF: left ventricular ejection fraction
TTE: Transthoracic echocardiography
